# Chemometric Optimization of Biologically Active Compounds Extraction from Grape Marc: Composition and Antimicrobial Activity

**DOI:** 10.3390/molecules27051610

**Published:** 2022-02-28

**Authors:** Aliona Ghendov-Mosanu, Daniela Cojocari, Greta Balan, Antoanela Patras, Ildiko Lung, Maria-Loredana Soran, Ocsana Opriş, Elena Cristea, Rodica Sturza

**Affiliations:** 1 Department of Oenology and Chemistry, Food Technology, Faculty of Food Technology, Technical University of Moldova, 9/9 Studentilor St., MD-2045 Chisinau, Moldova; cristea.ele@gmail.com (E.C.); rodica.sturza@chim.utm.md (R.S.); 2 Department of Microbiology and Immunology, Faculty of Medicine No I, “NicolaeTestemitanu State” University of Medicine and Pharmacy, 165 Stefan cel Mare Bd., MD-2004 Chisinau, Moldova; daniela.cojocari@usmf.md (D.C.); greta.balan@usmf.md (G.B.); 3 Department of Sciences, Faculty of Horticulture, “Ion Ionescu de la Brad” Iasi University of Life Sciences, 3 Mihail Sadoveanu Alley, 700490 Iasi, Romania; apatras@uaiasi.ro; 4 Department of Physics of Nanostructured Systems, National Institute for Research and Development of Isotopic and Molecular Technologies, 400293 Cluj-Napoca, Romania; ildiko.lung@itim-cj.ro (I.L.); loredana.soran@itim-cj.ro (M.-L.S.); ocsana.opris@itim-cj.ro (O.O.)

**Keywords:** grape marc, extraction parameters, biologically active compounds, mathematical models, antimicrobial activity, pathogenic microorganisms

## Abstract

The article focuses on the optimization of the extraction process of biologically active compounds (BAC) from grape marc—a by-product of the wine industry. The influence of temperature, specifically 30 °C, 45 °C and 65 °C, and ethanol concentration in solutions, specifically 0–96% (*v*/*v*) on the extraction yield of polyphenols, flavonoids, tannins and anthocyanins, were investigated. The composition of individual polyphenols, anthocyanins and organic acids, antioxidant activity (DPPH and ABTS) and CIELab chromatic characteristics of the grape marc extracts (GME), were characterized. The microbiostatic and microbicidal effects in direct contact of GME with pathogenic microorganisms, *Bacillus subtilis*, *Staphylococcus aureus*, *Escherichia coli*, *Klebsiella pneumoniae*, were determined in vitro. The influence of extraction parameters on the total polyphenol content (TPC), total flavonoid content (TFC), tannin content (TC), total anthocyanin content (TAC) and their interdependencies were studied using information analysis. A mathematical model was developed on cubic spline functions. The analysis of individual compounds showed the presence of a wide range of flavonoids (procyanidin B2, procyanidin B1, hyperoside and quercetin), flavones (catechin), hydroxybenzoic acid derivatives (gallic, protocatechuic, *p*-hydroxybenzoic acids, *m*-hydroxybenzoic acid, syringic acid), hydroxycinic acid derivatives and ferulic acid methyl ester. The malvidol-3-glucoside was the main anthocyanin identified in the extract. A high amount of tartaric acid was also found. GME showed significant antimicrobial activity against Gram-positive bacteria and lower activity against Gram-negative bacteria.

## 1. Introduction

Alcoholic and non-alcoholic beverage production generates waste and by-products that can be recovered. This would not only minimize their disposal costs and environmental hazards, but also add value to the development of new products. Traditional methods of using waste as fertilizer or animal feed use only a small part of the waste and are often not very effective [1]. Efforts must also be made to isolate and structurally elucidate new bioactive compounds. This will lead to achievements in the recovery of bioactive molecules, important for the development of innovative products, but it will also contribute to reducing environmental pollution [2]. A significant amount of residues is generated by the processing of grapes, among them, grape marc [3]. These residues are generally undervalued and used in animal feed (with low nutritional value), turned into fertilizer and even dumped in the environment, generating other problems, i.e., increased soil acidity, phytotoxicity, methane gas production, etc. [4]. Grape marc can become a product with potential economic profitability because it is a source of BAC (phenolic compounds, fatty acids, pectins, etc.) that can be used in the manufacture of food, cosmetics, dyes, supplements [5,6,7,8].

Numerous studies have shown the beneficial effects of polyphenols in grapes or wine on human health [9,10]. The general compositions of some grape marc have also been described [11,12]. Grape marc contains components that inhibit the proliferation of Caco-2 and HT-29 cancer cells by triggering apoptosis, has strong free radical scavengers and may provide some level of protection against certain cancers [13]. The profiles of phenolic compounds, recovered from waste from various wineries, were dominated by gallic acid, catechin and epicatechin. In addition, hydroxytyrosol, tyrosol, cyanidin glycosides and various phenolic acids, such as caffeic, procathechinic, syringic, vanillic, *o*-coumaric, *p*-coumaric acid, have also been identified [14]. A significant content of polyphenols (199.31 ± 7.21 mg gallic acid equivalents (GAE)/g), high antioxidant activity (cupric reducing antioxidant capacity test (CUPRAC)- 1036.98 mg trolox equivalents (TE)/g), enzyme inhibition (α-tyrosinase:151.30 ± 1.20 mg kojic acid equivalents (KAE)/g), is attested. The anti-inflammatory activity, as well as the antimicrobial activity of grape skin decoction, is higher than that reported for wine [15,16]. The extracts remarkably inhibit glucosyltransferases B and C (70–85% inhibition). Glycolytic decrease in pH can be attributed to partial inhibition of F-type adenosine triphosphate (F-ATP) activity (inhibition 30–65% at 125 µg/mL).

The biological activity of fermented marc is either as effective or significantly better than grape extracts [17]. Many phenolic compounds show significant antibacterial activity [18]. This is of particular interest for the development of natural alternatives to synthetic food preservatives and cosmetic applications [19,20]. Phenolic grape extracts, especially from different types of grape marc, are very effective against the specific virulence traits of *Streptococcus mutans*, despite major differences in their phenolic content. The mechanisms of antibacterial action of phenolic compounds are not yet fully deciphered, but it is known that these compounds involve many sites of action at the cellular level [21]. Several authors have explained this activity by the change of the permeability of cell membranes, the modification of the various intracellular functions induced by hydrogen binding of phenolic compounds to enzymes or by the changing of the rigidity of the cell wall, which leads to loss of integrity [22,23]. Polyphenols can induce irreversible damage to the cytoplasmic membrane, coagulation of cell contents and inhibition of intracellular enzymes. Tannins induce damage to the cell membrane, while phenolic acids can disrupt membrane integrity, causing leakage of essential intracellular constituents [24,25]. Flavonoids can bind to the cell walls of bacteria, promoting the formation of complexes, inhibit energy metabolism, DNA and RNA synthesis, intracellular changes in pH and interference with ATP [26,27].

Given the chemical composition of the grapes—and grape marc is obviously influenced by environmental factors and grape varieties [28,29,30,31]—extraction techniques should be optimized according to the composition of the pomace and directions for subsequent use of the extracts.

Several techniques are used to recover polyphenols from wine by-products, such as conventional solvent extraction, also called solid–liquid extraction (SLE), which is the most applied technique from an industrial point of view [32]. Several solvents have been studied for the extraction of polyphenols, but the preferred systems for food, pharmaceutical or cosmetic applications are those based on water and ethanol [33]. New unconventional techniques have emerged that can reduce extraction time, process temperature and solvent consumption, thus contributing to higher extraction efficiency and lower energy consumption. Some of the most relevant technologies are: ultrasonic-assisted extraction (UAE) [6,34], microwave-assisted extraction (MAE) [35,36], supercritical fluid extraction (SFE) [37], liquid pressure extraction (PLE) [38], ohmic heating (OH) [39], pulsed electric fields (PEF) [40,41] and enzyme-assisted extraction (EAE) [42]. Some enzymes, such as cellulases, hemicellulases, pectinases or amylases, can break down or weaken cell walls, releasing cytoplasmic contents (e.g., phenolic compounds) into the extraction solvent and thus improving extraction recovery. EAE can also be combined with other extraction techniques, such as EAU, MAE, PLE or SFE [43].

The optimization of the extraction parameters is easy to obtain in reproducible conditions, but the non-uniformity of by-products requires the presence of flexible solutions, easily adaptable to the composition of the extraction matrix. Response surface methodology (RSM) and artificial neural network (ANN) were used to model and optimize the extraction of polyphenolic compounds [44,45]. Statistical indicators have demonstrated the superiority of ANN. The comparison of different models of prediction of total polyphenols was performed by three mathematical equations: Spiro, Peleg and logarithmic, and two data extraction techniques: multivariate adaptive regression splines (MARS) and artificial neural network (ANN). The obtained results show that the data-mining techniques (MARS and ANNs) allow the creation of fast models and simple application, with a very good acceptability (coefficients of determination over 0.99) [45].

The aim of this article was to optimize the process of extracting bioactive compounds from red grape pomace according to temperature and solvent concentration, to model the interdependencies between extraction parameters by chemometric approach and to characterize the composition of extracts, antioxidant capacity and antimicrobial activity for subsequent use of these extracts in the food industry.

To optimize the extraction process, the polynomial spline functions were applied, which allows a division of the entire space of each independent variable into different sub-regions. Subsequently, truncated spline functions on two sides were defined as basic functions for describing the relationships between dependent and prediction variables in each distinct interval of the prediction variable. This model allows the adaptation of the extraction process to the fluctuating conditions of the composition of the grape marc solid fraction. For the solid–liquid extraction, the classic model was applied, applicable in the conditions of small grape processing enterprises, without additional expenses in terms of sophisticated equipment.

## 2. Results

### 2.1. The Influence of Temperature on the Extraction Yield of Bioactive Compounds

The influence of temperature, i.e., 30, 45 and 65 °C on the extraction yield of the TPC, TFC, TC and TAC in GME was investigated. The ethanolic solutions in the range of concentrations 0–96% (*v*/*v*) were used as solvents (Table 1).

The experimental data in Table 1 show that in grape marc ethanolic extracts, as the extraction temperature increases from 30 °C to 65 °C, the content of BAC increases with the variation of the hydroalcoholic concentration up to 60% (*v*/*v*) and then decreases to 96% (*v*/*v*). At 65 °C, the maximum values of the BAC content were attested for hydroalcoholic solutions of 60% (*v*/*v*). Thus, the TPC was 11.02 mg gallic acid equivalent (GAE)/g DW; TFC—7.76 mg GAE/g DW; TC—1.37 mg tannic acid equivalent (TAE)/g DW; and TAC—0.97 mg malvidin-3-glucoside equivalent (ME)/g DW. Minimum values of BAC content were obtained at a temperature of 30 °C and a concentration of the ethanolic solution of 96% (*v*/*v*), where the TPC was 1.37 mg GAE/g DW; TFC—0.84 mg GAE/g DW; TC—0.11 mg TAE/g DW; and TAC—0.23 mg ME/g DW.

The variation of the temperature from 30 to 65 °C in hydroalcoholic solutions with 60% (*v*/*v*) increases the extraction yield of BAC as follows: the TPC—1.47 times; the TFC—1.59 times; TC—1.63 times; and TAC—1.45 times.

**Figure 1:**
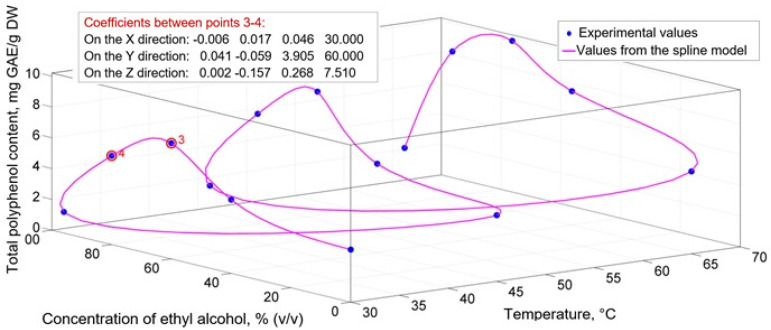
Mathematical model for the total content of polyphenols in grape marc extracts at different temperatures and concentrations of ethanolic solution, developed on cubic spline functions.

Cubic spline functions, which provide mathematical descriptions with variable coefficients, were used to model the extraction process. Figure 1 shows a mathematical model of the type TPC = f (t, C) for GME, using cubic spline functions. As can be seen, the modeling accuracy is maximum. The mathematical model curve goes through all 15 experimental points. For each interval between two points, different coefficients are obtained (in Figure 1 between experimental points 3 and 4), and the number of coefficients is represented by the degree of the polynomial. For a cubic spline function (k = 3), four coefficients are presented for each interval (k + 1 = 4) and for the three directions (x, y and z, which in this case represent the temperature (t), the concentration of the ethanolic solution (C) and the total polyphenol content (TPC). The results for the 15 experimental values are 14 groups of four coefficients obtained for each of the three directions, rendering (15 − 1) × 3 × 4 = 14 × 3 × 4 = 42 × 4 = 168 coefficients in total.

**Figure 2:**
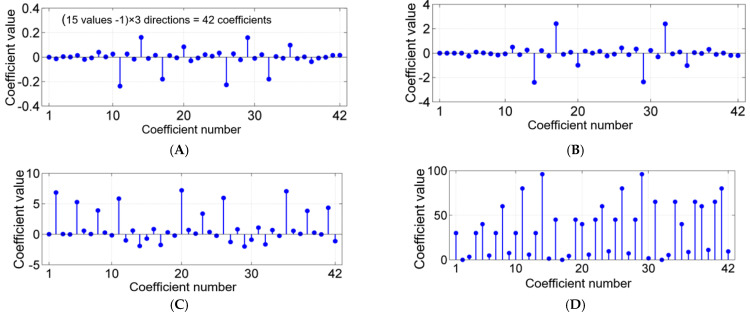
Coefficients of the mathematical model for TPC values in GME at different temperatures and concentrations of the hydroalcoholic solution, based on the cubic spleen functions: (**A**) coefficient c_1i_; (**B**) coefficient c_2i_; (**C**) coefficient c_3i_; (**D**) coefficient c_4i_.

Figure 2 shows the values of the four groups of coefficients of the cubic spline functions used for this mathematical model.

**Figure 3:**
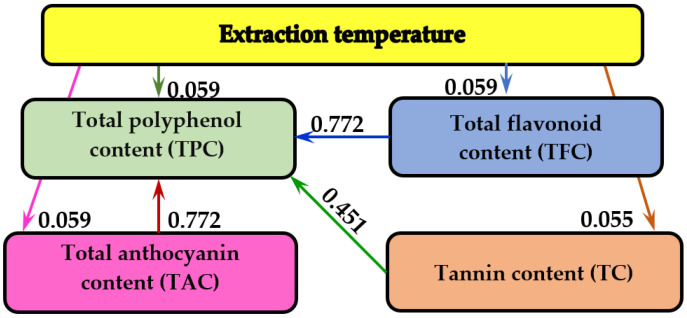
Analysis of mutual information on the influence of extraction temperature on the TPC, TFC, TAC and TC in GME.

The influence of extraction temperature on the TPC, TFC, TC and TAC in GME was studied using information analysis (Figure 3).

It was shown that the extraction temperature has the greatest influence on TPC, TPF and TAC (0.059 bits), followed by TC (0.055 bits). It was also found that the interdependence between TPC–TAC and TPC–TFC (0.772 bits) is more than 13 times higher than the maximum dependence between temperature and TPC, TPF and TAC (0.059 bits).

### 2.2. Characterization of Grape Marc Extract

Table 2 shows the composition of polyphenols, anthocyanins and individual organic acids, antioxidant activity and chromatic parameters CIELab for the hydroethanol extracts obtained from grape marc at the optimal extraction conditions, namely, a concentration of the ethanolic solution of 60% (*v*/*v*) and temperature of 65 °C.

Significant amounts of procyanidin B2 (824.73 µg/100 g DW), gallic acid (104.84 µg/100 g DW), catechin (72.04 µg/100 g DW), procyanidin B1 (71.51 µg/100 g DW), ferulic acid (44.09 µg/ 100 g DW) and ferulic acid methyl ester (39.78 µg/100 g DW) were detected in GME. *m*-hydroxybenzoic and sinapic acids were identified in small amounts, 0.54 µg/100 g DW and 0.43 µg/100 g DW, respectively.

The data in Table 2 show that malvidol-3-glucoside (519.92 µg/100 g DW) is the main anthocyanin identified in GME, followed by malvidol-3-acetylglucoside (119.31 µg/100 g DW), peonidol-3-glucoside (83.42 µg/100 g DW), petunidol-3-glucoside (79.54 µg/100 g DW), dolphinidol-3-glucoside (51.41 µg/100 g DW) and malvidol-3-coumarylglucoside (49.47 µg/100 g DW). Other anthocyanins listed in Table 2 were identified in smaller quantities.

The organic acids present in the grape marc hydroalcoholic extract were quantified (Table 2). Tartaric acid was found to be present in significant amounts (4279 mg/100 g DW), followed by acetic acid (500 mg/100 g DW), citric acid (415 mg/100 g DW), malic acid (373 mg/100 g DW) and ascorbic acid (36 mg/100 g DW).

Antioxidant activity in the GME was measured by DPPH and ABTS tests, and the respective values were 15.09 mmol TE/100g DW and 18.67 mmol TE/100gDW (Table 2).

The CIELab color parameters of the GME were analyzed. It was found that the values of luminosity L* were 60.1, component a*—9.72, component b*—1.22, chromaticity C*—9.80 and the hue angle H*—7.2° (Table 2).

### 2.3. Antimicrobial Activity of Grape Marc Extract

The microbiostatic and microbicidal effects in direct contact of GME with pathogenic microorganisms were determined in vitro for *Bacillus subtilis*, *Staphylococcus aureus*, *Escherichia coli* and *Klebsiella pneumoniae* (Table 3). These pathogenic bacteria are among the most common causes of microbiological contamination of food.

As a result of the tests performed, it was found that GME has a pronounced antimicrobial activity against Bacillus subtilis and *Staphylococcus aureus*; the zone diameter of inhibition was 11.0 mm. Grape marc showed lower antimicrobial activity for *Escherichia coli* (9.0 mm) and *Klebsiella pneumoniae* (7.0 mm) (Table 3). GME showed significant antimicrobial activity against Gram-positive bacteria (*Bacillus subtilis*, *Staphylococcus aureus*) and lower activity against Gram-negative bacteria (*Escherichia coli*) (Table 3). In the case of *Bacillus subtilis* and *Staphylococcus aureus*, GME exerted similar inhibitory (MIC) and bactericidal (MBC) activities, recording concentrations of 7.81 mg/mL and 15.62 mg/mL. *Escherichia coli* was found to be less sensitive to GME (MIC = 62.5 mg/mL, MBC = 125 mg/mL). Grape marc was shown not to exert antimicrobial activity against *Klebsiella pneumoniae* (Gram negative).

## 3. Discussion

Temperature is an important factor that influences the extraction efficiency of polyphenolic compounds, which can minimize the energy cost of the extraction process. Temperature has a positive effect on the extraction of polyphenolic compounds from plant sources, which is explained by the higher solubility of polyphenols in the solvent, by increasing diffusivity of the extracted molecules, improving the mass transfer of plant matter and reducing the viscosity of the solvent [46,47,48]. Additionally, high temperature extraction leads to changes in the plant matrix, and heat increases the permeability of cell walls, facilitating the extraction process [49]. Temperature may have a greater influence on the extraction yield of BAC than the concentration of ethanol in the solution [50]. In addition, the reduction in extraction duration can decrease the negative effect of enzymatic activity [51]. Nonetheless, high temperatures can affect the stability of certain polyphenolic compounds because of reactions involving chemical and enzymatic degradation or thermal decomposition [52]. It has been found that temperature of the extraction process is the most important parameter that affects the modification of polyphenols [53].

The evolution of extraction yield of the studied polyphenolic compounds was associated with an increase in the global speed of the extraction processes, which led to an improvement of the transfer of substances in the solvent [54]. Water and ethanolic solutions at 65 °C contributed to a better rupture of cell walls, thus accelerating the diffusion process [55]. The increase in extraction temperature led to a decrease in the viscosity of the extracts, respectively, the breaking of intermolecular bonds of the vegetal components. The shape of the intermolecular bond is determined by the chemical composition of the extract [56]. Plant extracts, containing polyphenolic substances with ionized groups create additional forces of interaction–repulsion of molecules, which reduces the density of molecules [57]. As the temperature increases, the molecular units of high molecular weight compounds (polyphenols) are able to oscillate more strongly, as a result of which the viscosity of plant extracts is reduced. The increase in molecular diffusion rate was also due to an increase in the kinetic energy of the molecules and a decrease in the viscosity of plant extracts [58].

The data in Table 1 show that low extraction temperatures are less efficient in the extraction of polyphenolic compounds, due to their low solubility and the energy required to swell and disrupt the cell walls of grape pomace. Rajha et al. [59] optimized the extraction process of polyphenols, flavonoids and tannins from grape by-products at the extraction temperature of 93 °C, for 93 min, with ethanolic solution of 66% concentration (*v*/*v*). TPC extracted was 5.5 g GAE/100 g DW; TFC—5.4 g GAE/100 g DW; and TC—12.3 g/L. Bucić-Kojic et al. [60] investigated the influence of ethanolic solution concentration of 50, 70 and 96% (*v*/*v*) and extraction temperature of 25–80 °C for 200 min on TPC in Frankovka grape seeds and demonstrated that the best results were obtained at 80 °C and 50% (*v*/*v*) ethanolic solution concentration. Spino and De Faveri [61] established that the temperature of 60 °C is optimal for the extraction of polyphenolic compounds from Barbera red grape pomace.

The dependence of BAC at different concentrations of ethanolic solutions depending on the extraction temperature (Table 1) demonstrates the existence of direct or indirect dependencies on various portions. The existence of nonlinear dependencies leads to the need to establish mathematical models with the same character (Figure 1). Because of this, cubic spline functions were used, which provide mathematical descriptions with variable coefficients (Figure 2). Thus, any functional variation can be modeled with maximum precision [62]. Taofiq et al. [63] also used second-order polynomial mathematical models to evaluate the influence of ergosterol extraction parameters on *Pleurotus* mushrooms.

The informational analysis of the experimental data allowed us to establish the influence of temperature on the extraction yield of polyphenolic compounds during the experiments (Figure 3). It was shown that the extraction temperature had less influence on the measured parameters than the interdependence between polyphenolic compounds TAC–TPC and TFC–TPC, probably due to the short extraction time (90 min). The influence of pH and different salts (NaCl, CaCl_2_, KNO_3_) at different concentrations (0.001–0.1M) on the CIELab color parameters of rosehip extracts was studied by informational analysis [64].

The efficiency of the extraction process depends on the nature of the polyphenolic compounds present in the grape marc. Table 2 shows the composition of individual polyphenols identified by the HPLC method in hydroethanolic extracts from Merlot red grape marc at a concentration of 60% (*v*/*v*) ethanolic solution and at an extraction temperature of 65 °C. The flavonoids (procyanidin B2, procyanidin B1, hyperoside and quercetin), flavones (catechin), hydroxybenzoic acid derivatives (gallic, protocatechuic, *p*-hydroxybenzoic, *m*-hydroxybenzoic, syringic), hydroxycinic acid derivatives (ferulic and sinapic) and methyl ester were identified. 

Hydroxybenzoic and hydroxycinnamic acid derivatives have been identified in red grape pomace *Vitis vinifera* L. [65]. Anastasiadi et al. [66] identified gallic acids in grape stems and seeds, and syringic acid in stems. Gallic, *p*-coumaric and coutaric acids, catechin, epicatechin, resveratrol and quercetin have been detected in Cabernet grape pomace [67]. Quercitin, kemferol, catechin, epicatechin, *trans*-resveratrol and gallic acid have been identified in grape marc of Cabernet Sauvignon and Merlot (*Vitis vinifera* L.), Bordeaux and Isabell (*Vitis labrusca* L.) from Brazil [68].

A selective extraction of anthocyanins was demonstrated in this study (Table 2). Monoglycosides were extracted more efficiently compared to acetylated glycosides and coumarin glycosides. The polarity and stability of anthocyanins were probably influenced by methoxyl and hydroxyl groups [69]. Malvidol was extracted in larger quantities than peonidol, followed by petunidol, delfinidol and cyanidol. Other bibliographic sources attest that malvidol-3-O-glucoside, peonidol-3-O-glucoside and petunidol 3-O-glucoside have been identified in grape marc in increased quantities [65,70].

Grape marc has been shown to contain high amounts of tartaric acid and low amounts of ascorbic acid. Tartaric acid was the main acid identified in the studied grape marc [71]. The literature shows that the content of organic acids in plant extracts is influenced by the variety of plant matter, degree of ripeness, growth region, level of insolation and climatic conditions [72,73].

The antioxidant activity in grape marc extract was measured by the DPPH and ABTS tests. Several bibliographic sources attest that the antioxidant activity is influenced by the presence of polyphenolic compounds [74,75]. It is difficult to compare the antioxidant activity of grape marc extracts with bibliographic sources because different analytical methods, reference standards, units of measurement and different grape marc samples have been applied. Antioxidant activity may also be influenced by the geographical origin of the grapes and the method of extraction of bioactive compounds [76]. Our results were compared with data published by Negro et al. [70], who evaluated the antioxidant activity by the DPPH test in three varieties of grape pomace from Italy. The antioxidant activity values were shown to range from 122 µmol TE/g DW (Malvasia di Lecce variety) to 251 µmol TE/g DW (Primitivo variety).

The extract color is determined by the presence of anthocyanins in the grape skin, being an important feature because it can be used as a natural dye in the food industry. The CIELab color parameters of GME demonstrate the prevalence of red pigments (a* = 9.72) and the low amount of yellow pigments (b* = 1.22). In accordance with the hue angle (H* = 7.2°), the grape marc extract is in the first trigonometric quadrant, in which the red tone predominates. Several authors attest that the intensity of the extract color depends not only on the total content of anthocyanins, but also on the chemical structure of anthocyanins, extraction conditions, the presence of enzymes, oxygen, etc. [77,78].

The antimicrobial potential of grape marc is attributed to the content of polyphenolic compounds. Polyphenolic compounds from grape marc have been shown to have a significant influence on examined Gram-positive bacteria (*Bacillus subtilis* and *Staphylococcus aureus*) compared to Gram-negative bacteria (*Escherichia coli* and *Klebsiella pneumoniae*). The same results were reported by Kabir et al. [79]. Antimicrobial activity of polyphenols may involve such mechanisms as destabilization and permeability of the cytoplasmic membrane and inhibition of the enzyme by oxidized products, thus inhibiting a synthesis of nucleic acids by Gram-negative and Gram-positive bacteria [80]. Gallic acid can alter bacterial hydrophobicity, while quercetin leads to bacteriostasis by damaging cell walls and membranes [24,81]. Other authors reported that marc extracts and grape seeds, containing flavonoids and their derivatives, showed antimicrobial activity against Gram-positive bacteria, such as *Staphylococcus aureus*, *Bacillus cereus*, *Bacillus subtilis*, *Bacillus coagulans*, *Listeria monocytogenes* and Gram-negative *Escherichia coli*, *Pseudomonas aeruginosa* [82,83]. Anthocyanins may also be involved in enhancing the antimicrobial activity of grape marc [84]. Antimicrobial activity of grape marc can be influenced by the number of hydroxyl groups and the degree of polymerization of phenolic compounds [85]. Olech et al. [86] reported on the correlation between polyphenol content, antioxidant capacity and antibacterial potential of vegetable extracts.

## 4. Materials and Methods

### 4.1. Materials

Red grape marc (*Vitis vinifera* L.) of the “Merlot” variety was obtained from the Pilot Laboratory of Microvinification at the Technical University of Moldova Chisinau, Republic of Moldova). The Folin–Ciocalteu reagent, tannic acid, acetonitrile and formic acid were purchased from Merck (Darmstadt, Germany); (+)-catechin (98%), quercetin, syringic acid, ferulic acid, gallic acid (98%), protocatechuic acid, parahydroxybenzoic acid, salicylic acid (99.9%), ferulic acid methyl ester, DPPH, tartaric acid, ascorbic acid, citric acid (99.5%) and acetic acid (99.8%) were obtained from Sigma-Aldrich (Darmstadt, Germany; Tokyo, Japan; Shanghai, China). Sinapic acid (98%) was purchased from Alfa Aesar (Kandel, Germany). Procyanidin B1, procyanidin B2 and hyperoside were purchased from Extrasynthese (Genay, France). Quercetin (>95%) was obtained from Sigma-Aldrich (Bangalore, India). Cyanidin 3-glucoside chloride (≥98%), peonidin 3-glucoside chloride (≥90%), malvidin 3-glucoside chloride (≥95%) and malvidin 3,5-diglucoside chloride (≥90%) were obtained from PhytoLab, (Vestenbergsgreuth, Germany). All spectrophotometric measurements were performed on the Analytik Jena Specord 200 Plus (Jena, Germany) spectrophotometer.

### 4.2. Extract Characterization

The extraction was performed in ethanol at various concentrations 0%, 40%, 60%, 80% and 96% (*v*/*v*) (1:8 ratio) under stirring at 60 rpm for 90 min at 30 °C, 45 °C and 65 °C [87]. After filtration, the total content of polyphenols, flavonoids, tannins and anthocyanins was determined. The extract was stored in glass bottles under refrigeration in the dark.

#### 4.2.1. Total Polyphenols and Flavonoids by Folin–Ciocalteu

The method described by [88] was used to determine the total polyphenol content. The results were calculated from a calibration curve using gallic acid (0–500 mg/L) and expressed in equivalents of gallic acid per 1 g of dried weight (DW) of grape marc extract (mg GAE/g DW). The method described by Spranger et al. [89] was used to determine the total flavonoid content, which was calculated by measuring the difference between the total polyphenol content until and after the precipitation of flavonoids with formaldehyde in a strong acidic medium. The results were expressed in mg GAE/g DW.

#### 4.2.2. Total Tannins by Folin–Ciocalteu

The tannin content was determined using the Folin–Ciocalteu reagent, a method described by Waterman and Mole [90]. The results were calculated from a calibration curve using tannic acid (0–50 mg/L) and expressed in equivalents of tannic acid per 1 g of dried weight (DW) of grape marc extract (mg TAE/g DW).

#### 4.2.3. Total Anthocyanins

To determine the total anthocyanin content, a method described by Giusti and Wrolstad [91] was used. The results were expressed in equivalents of malvidin-3-glucoside per 1 g of dried weight (DW) of grape marc extract (mg ME/g DW).

#### 4.2.4. HPLC Analysis of Polyphenols

The content of individual polyphenols in the ethanolic grape marc extract of 60% (*v*/*v*) at a temperature of 65 °C was analyzed using the Agilent 1100 Series HPLC (Santa Clara, CA, USA). The gradient was optimized using trifluoroacetic acid (TFA) as an eluent acidification of 1% CH_3_OH (A channel) and 50% CH_3_OH (B channel) acidified to 2.15 pH with TFA. The column system was composed of a pre-column SecurityGuard ULTRA Cartridges HPLC (Torrance, CA, USA) C18 for 4.6 mm ID coupled with a Kinetex 5 µm C18 100 Å 250 × 4.6 mm column manufactured by Phenomenex at 35 °C. A run time of 90 min and an injection volume of 20 μL were used. The phases were A: H_2_O:CH_3_OH (99:1) and B: H_2_O:CH_3_OH (50:50), with a flow of 1.5 mL/min. The detection was carried out at 256, 280, 324 and 365 nm. The gradient of elution was 100% (A): for 10 min; 82% (A): 18% (B) for the next 10 min; 70% (A): 30% (B) for 10 min; 65% (A): 35% (B) for 6 min; 40% (A): 60% (B) for 15 min; 20% (A): 80% (B) for 5 min; 100% (B) for 15 min and 100% (A) for 10 min. The content of specific polyphenols was determined by comparison of retention times and peaks of the sample chromatogram with ones from the chromatogram of synthetic standards listed in Table 4.

#### 4.2.5. HPLC Analysis of Anthocyanins

The content of individual anthocyanins in the ethanolic grape marc extract of 60% (*v*/*v*) at a temperature of 65 °C was analyzed by direct separation by HPLC Agilent 1100 Series HPLC (Santa Clara, CA, USA), using reverse phase column with gradient elution by water/formic acid/acetonitrile with detection at 518 nm [92]. The identification of anthocyanins from grape marc samples was carried out by the comparison of UV–VIS spectra and retention times of the sample peaks with those of the standard solutions (Table 5).

#### 4.2.6. Quantification of Organic Acids

The quantification of organic acids was performed in the ethanolic grape marc extract of 60% (*v*/*v*) at a temperature of 65 °C. The Agilent 7100 CE System (Santa Clara, CA, USA) and the method described by Cristea et al. [93] were used. The total organic acid content was expressed in mg/100 g DW of grape marc extract.

#### 4.2.7. Antioxidant Activity by Reaction with DPPH Radical

The antiradical DPPH activity of ethanolic grape marc extract of 60% (*v*/*v*) at a temperature of 65 °C was measured following the method described by Brand-Williams et al. [94]. The results were expressed in mmol trolox equivalents per 100 g of dried weight (DW) of grape marc extract (mmol TE/100 g DW) after the calibration curve (0–250 µmol/L) created using trolox as standard.

#### 4.2.8. Antioxidant Activity by Reaction with ABTS Radical

The antiradical ABTS activity of the ethanolic grape marc extract of 60% (*v*/*v*) at a temperature of 65 °C was determined according to the method described by Re et al. [95]. The results were expressed as mmol TE/100 g DW after the calibration curve (0–2000 µmol/L) with trolox.

#### 4.2.9. Color Parameters (CIELab)

WinASPECT PLUS software (Jena, Germany) and a Specord 200 Plus spectrophotometer (Jena, Germany) were used to evaluate the color parameters (CIELab). Luminosity (L*), red/green component (a*), yellow/blue component (b*), chromaticity (C*) and hue angle (H*) are presented as results. These parameters were measured following the official method [96].

### 4.3. Analysis of Antimicrobial Activity

#### 4.3.1. Test Microorganisms

Microbial strains of *Staphylococcus aureus* ATCC 25923, *Bacillus subtilis* ATCC 6633, *Escherichila coli* ATCC 25922 and *Klebsiella pneumoniae* ATCC 13883 were used to test the effectiveness of extracts. Standard bacterial cultures were offered by the Microbiology and Immunology Department, Nicolae Testemitanu State University of Medicine and Pharmacy (Chisinau, Republic of Moldova).

#### 4.3.2. Agar Well Diffusion Method

Ethanolic extracts from grape marc obtained at 60% (*v*/*v*) and dealcoholized by rotary evaporator HL/G3 Heidolph (Schwabach, Germany) were used. Agar well diffusion method is widely used to evaluate the antimicrobial activity of plant extracts. The agar plate surface was inoculated by spreading a volume of the microbial inoculum over the entire agar surface. Then, a hole with a diameter of 6 to 8 mm was punched aseptically with a sterile cork borer, and a volume (100 µL) of the antimicrobial extract solution of desired concentration was introduced into the well. Then, agar plates were incubated under suitable conditions for each test microorganism. The antimicrobial agent diffuses in the agar medium and inhibits the growth of the tested microbial strain. After the incubation period, the result was read. The diameter of the inhibition zone, which marks the absence of microbial growth, was measured with the shubler ruler [97,98].

#### 4.3.3. Minimal Inhibitory Concentration (MIC) and Minimum Bactericidal Concentration (MBC) Determination

Minimum inhibitory concentration values were studied for the bacterial strains sensitive to the extracts in the broth macro-dilution method. The two-fold dilution method was performed using as many as 10 test tubes. First, a pipette was used to dispense 2 mL of broth medium to each test tube. Then, as much as 2 mL of the extract was placed in tube number 1 with a concentration of 50%. Afterward, the same pipette was used to transfer 2 mL of extract from test tube number 1 into test tube number 2, then diluted in the broth medium inside test tube number 2 to make the concentrated extract of 25%. This series was the first stage of two-fold dilutions. Then, each tube was inoculated with a microbial inoculum prepared in saline solution after dilution of a standardized microbial suspension adjusted to 0.5 McFarland scale. After well mixing, the inoculated tubes were incubated (mostly without agitation) under suitable conditions for each tested microorganism. MBC was determined after sub-culturing a sample from the tubes, yielding a negative microbial growth after incubation on the surface of non-selective agar plates to determine the number of colonies (CFU/mL) after 24 h of incubation. The bactericidal endpoint (MBC) was subjectively defined as the lowest concentration at which 99.9% of the final inoculum is killed. Similar tests were performed simultaneously for growth control (broth + inoculum) and sterility control (broth + test sample) [99].

### 4.4. Mathematical Modeling

Mathematical modeling (polynomial mathematical model and information analysis of experimental data) was performed in the MATLAB program (MathWorks, Inc., Natick, MA, USA). Cubic spline functions were used, and the coefficients of the mathematical model were determined [62,100]. For the spline function of order *k*, the interpolation polynomial for any size *x* with *n* discrete values has the form

(1) $$P_{i}(x)={\sum_{j=1}^{k+1}\left(x-\xi_{i}\right)}^{i-k}c_{ji}\;;\;i=1\ldots n-1$$

with the interval between two points *ξ_i_* and the local polynomial coefficients *c_ji_*. Therefore, at each interval between two points, different coefficients are obtained, and the number of coefficients is given by the degree of the polynomial that defines the spline function. The spline functions provide a virtually zero modeling error that is demonstrated by the corresponding curve passing through each point on the graph.

Information analysis allows the evaluation of mutual influences among determined parameters. It is based on two main concepts: information and entropy. Information is a fundamental concept in prediction and is characterized by a distribution of probabilities, and entropy is a product of probability and information on all events. Entropy is the quantitative measure of uncertainty. Thus, the higher the values of mutual information, the lower the uncertainties [101]. Parameter names are given in the rectangles of the graph, and the values of mutual information, measured in bits, are mentioned on the arrows of the graph.

### 4.5. Statistical Analysis

All calculations were performed using Microsoft Office Excel 2007 (Microsoft, USA). Data obtained in this study are presented as mean values ± the standard error of the mean calculated from three parallel experiments. The comparison of average values was based on the one-way analysis of variance (ANOVA) according to Tukey’s test at significance level *p* ≤ 0.05, using the Staturphics program Centurion XVI 16.1.17 (Statgraphics Technologies, Inc., The Plains, VI, USA).

## 5. Conclusions

The extraction yield of bioactive compounds was influenced by the polarity and viscosity of the hydroalcoholic extract, increasing in the range 0–60% (*v*/*v*) and decreasing to 96% (*v*/*v*). Increasing the temperature from 30 °C to 65 °C led to an increase in the extraction yield of TPC, TFC, TC and TAC from grape marc.

The dependence of BAC content in different hydroalcoholic concentrations and extraction temperatures was characterized by nonlinear dependencies. This led to the need to model extraction processes based on cubic spline functions, which provide mathematical descriptions with variable coefficients and maximum accuracy.

Informational analysis of the experimental data allowed the ascertainment of the influence of temperature on extraction yield of polyphenolic compounds. It was shown that extraction temperature had less influence on the directly measured parameters than the interdependence between the polyphenolic compounds TAC–TPC and TFC–TPC.

The composition of individual polyphenols in GME was established. Selective extraction of anthocyanins was demonstrated. Monoglycosides were better extracted than acetylated glycosides and coumarin glycosides. Malvidol was extracted in larger quantities than peonidol, followed by petunidol, delfinidol and cyanidol.

GME was characterized by an important antioxidant activity, which was determined by the DPPH and ABTS tests being 15.09 mmol TE/g DW and 18.67 mmol TE/100 g DW, respectively. The chromatic parameters of GME demonstrated the prevalence of red pigments (9.72) and the low amount of yellow pigments (1.22), which is an important feature because it can be used in the development of natural dyes in the food industry.

GME was shown to have a significant influence on Gram-positive bacteria (*Bacillus subtilis* and *Staphylococcus aureus*) compared to Gram-negative bacteria (*Escherichia coli* and *Klebsiella pneumoniae*).

The obtained results showed that the application of solid–liquid extraction methods allows extracts rich in polyphenolic compounds with antioxidant capacity and antimicrobial potential to be obtained without the application of technologies that would require expensive equipment and consumables. The extraction can be carried out directly at the wineries, after the processing of the grapes, using the alcohol obtained by distilling the pomace and the wine yeasts used. The application of modeling based on cubic spline functions allows the optimization of the extraction according to the available ethanol concentration.

## Figures and Tables

**Table 1:** Influence of temperature on the extraction yield of the TPC, TPF, TC and TAC in GME depending on the concentration of ethanolic solutions (the results are expressed as means ± standard deviations of three experiments).

Concentration of Ethanolic Solution, % (*v*/*v*)	Temperature, °C		
	30	45	65
Total Polyphenol Content (TPC), mg GAE/g DW			
0	3.41 ± 0.21 ^c^	4.30 ± 0.14 ^d^	5.35 ± 0.18 ^e^
40	4.78 ± 0.18 ^d^	5.77 ± 0.22 ^e^	8.69 ± 0.17 ^g^
60	7.51 ± 0.25 ^f^	9.52 ± 0.24 ^h^	11.02 ± 0.02 ^i^
80	5.76 ± 0.18 ^e^	7.14 ± 0.13 ^f^	9.40 ± 0.10 ^h^
96	1.37 ± 0.11 ^a^	1.73 ± 0.11 ^a^	2.39 ± 0.10 ^b^
Total Flavonoid Content (TFC), mg GAE/g DW			
0	1.83 ± 0.03 ^b^	2.50 ± 0.03 ^b,c^	3.32 ± 0.04 ^c,d^
40	3.04 ± 0.12 ^c^	3.85 ± 0.11 ^d^	5.98 ± 0.08 ^g^
60	4.89 ± 0.09 ^e^	6.87 ± 0.07 ^h^	7.76 ± 0.14 ^i^
80	4.31 ± 0.10 ^e^	5.43 ± 0.05 ^f^	7.38 ± 0.15 ^h^
96	0.84 ± 0.03 ^a^	0.94 ± 0.04 ^a^	1.48 ± 0.04 ^a^
Tannin Content (TC), mg TAE/g DW			
0	0.27 ± 0.02 ^c^	0.35 ± 0.04 ^c,d^	0.53 ± 0.02 ^e^
40	0.47 ± 0.02 ^d^	0.57 ± 0.04 ^e^	1.16 ± 0.04 ^i,j^
60	0.84 ± 0.03 ^g^	1.11 ± 0.07 ^i,j^	1.37 ± 0.01 ^k^
80	0.74 ± 0.05 ^f,g^	0.95 ± 0.04 ^h^	1.24 ± 0.04 ^j,k^
96	0.11 ± 0.01 ^a^	0.14 ± 0.02 ^a,b^	0.18 ± 0.01 ^b^
Total Anthocyanin Content (TAC), mg ME/g DW			
0	0.02 ± 0.01 ^a^	0.03 ± 0.01 ^a^	0.05 ± 0.01 ^a^
40	0.35 ± 0.01 ^c^	0.56 ± 0.01 ^e^	0.71 ± 0.01 ^f,g^
60	0.67 ± 0.01 ^f^	0.79 ± 0.01 ^g^	0.97 ± 0.02 ^h^
80	0.45 ± 0.01 ^d^	0.62 ± 0.01 ^f^	0.79 ± 0.01 ^g^
96	0.23 ± 0.01 ^b^	0.29 ± 0.01 ^b,c^	0.38 ± 0.01 ^c^

Different letters (^a–k^) designate statistically different results (*p* ≤ 0.05).

**Table 2:** Polyphenols, anthocyanins and individual organic acids, antioxidant activity and CIELab color parameters in grape marc hydroethanolic extract at 60% (*v*/*v*) and extraction temperature of 65 °C (the results are expressed as means ± standard deviations of three experiments).

Indices	Quantity
Polyphenols	
Gallic acid, µg/100 g DW	104.84 ± 9.21
m-Hydroxybenzoic acid, µg/100 gDW	0.54 ± 0.07
Protocatechuic acid, µg/100 gDW	17.20 ± 0.65
p-Hydroxybenzoic acid, µg/100 g DW	18.28 ± 0.32
Syringic acid, µg/100 gDW	10.22 ± 0.17
Ferulic acid, µg/100 gDW	44.09 ± 1.06
Sinapic acid, µg/100 gDW	0.43 ± 0.09
Catechin, µg/100 gDW	72.04 ± 1.16
Quercetin, µg/100 gDW	10.22 ± 0.35
Hyperoside, µg/100 gDW	19.89 ± 0.50
Procyanidin B1, µg/100 gDW	71.51 ± 0.97
Procyanidin B2, µg/100 gDW	824.73 ± 13.26
Ferulic acid methyl ester, µg/100 g	39.78 ± 1.04
Anthocyanins	
Cyanidol-3-glucoside, µg/100 gDW	43.65 ± 1.87
Petunidol-3-glucoside, µg/100 gDW	79.54 ± 1.65
Dolphinidol-3-glucoside, µg/100 gDW	51.41 ± 1.23
Peonidol-3-glucoside, µg/100 gDW	83.42 ± 2.02
Malvidol-3-glucoside, µg/100 gDW	519.92 ± 14.65
Peonidol-3-acetylglucoside, µg/100 g DW	15.52 ± 0.48
Malvidol-3-acetylglucoside, µg/100 gDW	119.31 ± 9.04
Peonidol-3-coumarylglucoside, µg/100 g DW	7.76 ± 0.83
Malvidol-3-coumarilglucoside, µg/100 g DW	49.47 ± 0.79
Organic acids	
Malic acid, mg/100gDW	373 ± 7
Citric acid, mg/100gDW	415 ± 5
Ascorbic acid, mg/100gDW	36 ± 1
Acetic acid, mg/100gDW	500 ± 3
Tartaric acid, mg/100gDW	4279 ± 81
Antioxidant activity	
Antioxidant activity (DPPH), mmol TE/100gDW	15.09 ± 1.72
Antioxidant activity (ABTS), mmol TE/100gDW	18.67 ± 0.89
CIELab Chromatic Characteristics	
L*	60.10 ± 0.15
a*	9.72 ± 0.09
b*	1.22 ± 0.05
C*	9.80 ± 0.07
H*, °	7.2 ± 0.1

DPPH = 2,2-diphenyl-1-picrylhydrazyl-hydrate, ABTS = 2,20-azino-bis-3-ethylbenzthiazoline-6-sulphonic acid, TE = Trolox equivalents, L* = luminosity, a* = red/green component, b* = yellow/blue component, C* = chromaticity, H* = hue angle.

**Table 3:** The antimicrobial activity, minimum inhibitory concentration (MIC) and minimum bactericidal concentration (MBC) of GME against bacterial strains (the results are expressed as means ± standard deviations of three experiments).

Bacterial Strain	Zone Diameter of Inhibition, mm	MIC, mg/mL	MBC, mg/mL
*Bacillus subtilis* ATCC 6633	11 ± 2	7.81 ± 0.21	15.62 ± 0.62
*Staphylococcus aureus* ATCC 25923	11 ± 2	7.81 ± 0.19	15.62 ± 0.41
*Escherichia coli* ATCC 25922	9 ± 1	62.50 ± 1.57	125.00 ± 5.00
*Klebsiella pneumoniae* ATCC13883	7 ± 1	nd	nd

nd = no detected activity.

**Table 4:** Characteristics of polyphenol standards used in HPLC analysis and their retention times.

Compound	Max Absorption (nm)	Retention Time (min)
Gallic acid	280	5.294
Protocatechuic acid	256	9.267
*p*-hydroxybenzoic acid	256	13.918
Procyanidin B1	280	16.704
*m*-hydroxybenzoic acid	280	17.989
Catechin	280	18.53
Procyanidin B2	280	23.433
Syringic acid	280	25.002
Ferulic acid	324	36.233
Sinapic acid	324	38.564
Ferulic acid methyl ester	365	57.754
Quercetin	256	65.278

**Table 5:** Anthocyanins used as standards in HPLC analysis and their retention times.

**Compound**	Dolphinidol-3-glucoside	Cyanidol-3-glucoside	Petunidol-3-glucoside	Peonidol-3-glucoside	Malvidol-3-glucoside	Peonidol-3-acetylglucoside	Malvidol-3-acetylglucoside	Peonidol-3-coumarylglucoside	Malvidol-3-coumarilglucoside
**Retention Time (min)**	8.064	9.834	11.080	13.315	14.768	27.775	29.379	42.725	43.739

## Data Availability

Not applicable.

## Sample Availability

Not available.
